# Health Equity in Systematic Reviews: A Tutorial—Part 1 Getting Started With Health Equity in Your Review

**DOI:** 10.1002/cesm.70055

**Published:** 2025-10-30

**Authors:** Jennifer Petkovic, Jordi Pardo Pardo, Vivian Welch, Omar Dewidar, Lara J. Maxwell, Andrea Darzi, Tamara Lotfi, Lawrence Mbuagbaw, Kevin Pottie, Peter Tugwell

**Affiliations:** ^1^ University of Ottawa Ottawa Ontario Canada; ^2^ Bruyère Health Research Institute Ottawa Ontario Canada; ^3^ Temerty Faculty of Medicine Toronto Ontario Canada; ^4^ Department of Health Research Methods, Evidence & Impact McMaster University Hamilton Ontario Canada; ^5^ Department of Family Medicine Dalhousie University Halifax Nova Scotia Canada; ^6^ Department of Medicine University of Ottawa Ottawa Ontario Canada; ^7^ Ottawa Hospital Research Institute Methodological and Implementation Research Ottawa Ontario Canada; ^8^ School of Epidemiology and Public Health University of Ottawa Ottawa Ontario Canada

## Abstract

This tutorial focuses on how to get started with considering health equity in systematic reviews of interventions. We will explain why health equity should be considered, how to frame your question, and which interest‐holders to engage. This is the first tutorial in a series on health equity. The second tutorial focuses on implementing health equity methods in your review.

## Graphical abstract

Health‐equity‐1 micro learning module

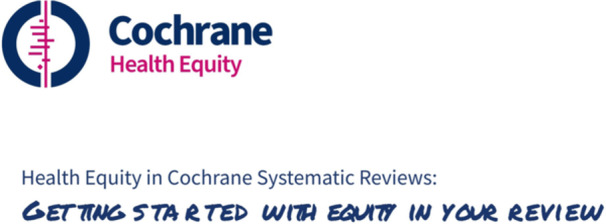

## 1 What Is Health Equity?

Health inequalities are differences in health outcomes between individuals or groups. While some differences may be expected due to biological variations, systematic, socially produced, and avoidable differences are considered unjust. These unjust differences are often referred to as health inequities. Health equity is achieved when everyone has the opportunity to attain their highest level of health [1, 2]. The social determinants of health (SDH) refer to the conditions in which a person is born, lives, and works, and include access to power, money, and resources [3] and impact the achievement of health equity. Understanding the SDH allows us to consider their potential effects on baseline risks of a problem or condition, and distributional effects on the intervention and its effectiveness.

The burden of most health conditions is disproportionately borne by people experiencing higher levels of adverse social determinants of health and those who are affected by systemic barriers to health opportunities across social and personal characteristics. The Campbell and Cochrane Health Equity Thematic Group uses PROGRESS‐Plus to systematically identify these factors and their relevance to a systematic review. PROGRESS stands for **P**lace of residence, **R**ace/ethnicity/culture/ancestry/language, **O**ccupation, **G**ender and sex, **R**eligion, **E**ducation, **S**ocioeconomic status, and **S**ocial capital [4]. The “Plus” allows for considerations of other context‐dependent factors, such as additional personal characteristics (e.g., age, disability), features of relationships (e.g., exclusion from school), and time‐dependent relationships (e.g., transition from pediatric to adult care) [5]. These factors and characteristics are described in Appendix A. Authors are urged to consider and report these characteristics separately, but, it is important to consider the presence of important interactions between characteristics, particularly when their combined effect could lead to unique experiences of health inequities. The concept of interactions and intersections between these factors is referred to as intersectionality. This concept was originally introduced by Black feminists to describe the interrelated effects of sexism, racism, classism, and homophobia [6]. It refers to the overlapping, additive or multiplicative effects that these experiences can have on health outcomes [7, 8, 9]. Considering intersectionality is not as simple as adding the effects of individual characteristics, such as gender, race, and socioeconomic status. Instead, it requires a clear focus on social power as a causal mechanism, and a relational approach to how power and privilege act in ways that are shaped by the interactions between different forms of oppression [10]. There is currently no operational guidance for assessing intersectionality within reviews, but authors should consider the possible effects of intersectionality when interpreting their findings.

## 2 Why Consider Health Equity in a Systematic Review?

Systematic reviews are used to inform health practice and policy. Decision‐makers want to know to whom the evidence applies and in which settings. Systematic review authors can support this decision‐making by considering the relevance of health equity from the outset, at the registration and protocol stages. Methods should be designed to assess the effects of the intervention on health equity. Authors should describe who is affected by the condition, who is included in the primary studies, the settings in which the studies took place, and then provide information that supports judgments regarding applicability.

## 3 How to Consider Health Equity in Systematic Reviews

Whether you are planning a protocol for a new review, already working on your review, or considering updating your published review, you can add a health equity dimension.

At a minimum, health equity reporting is an opportunity to reflect on the generalizability of the results, whether by population (e.g., those with different levels of education or income) or setting (for instance, high income vs low‐income countries). There may be important differences between specific populations experiencing health inequities such as: they might have different baseline risks, they might value outcomes differently, or they might have challenges using or accessing the intervention. Health equity reporting can facilitate future research recommendations (see Box 1).

When planning a review, there are six main ways to consider health equity. In this tutorial, we cover the first two to help you get started with your review. We describe the remaining four in part 2:

1. Decide which interest‐holders should be engaged in the systematic review process.
2. Consider health equity at the question formulation stage.
3. Decide what methods will be used to identify, extract, and appraise evidence related to health equity.
4. Describe populations using health equity characteristics.
5. Consider implications for “Summary of findings” tables (e.g., separate tables for disadvantaged populations, separate rows for differences in risk of events).
6. Interpret findings related to health equity in the discussion.

Box 1 **How to get started.**

Considering health equity in your protocol and review doesn't mean you need to completely change your review question. We are not suggesting that you identify and consider every possible population experiencing health inequities. Instead, systematic review authors are encouraged to use evidence‐informed and contextually grounded approaches, drawing on existing literature, theory, and lived experience to prioritize populations and guide research questions. You can include health equity without adding to your budget or timelines. For example, you can:

‒ Prioritize populations for whom the existing literature suggests the baseline risk of the condition may be different
‒ Prioritize populations for whom the existing literature suggests the mechanism of action of the intervention may be different
‒ Prioritize populations for whom access to the intervention may differ
‒ Extract and report data related to the populations included within the studies and whether they reflect the population experiencing the condition or problem of interest

## 4 Including Interest‐Holders and People With Lived‐Experience

### 4.1 Getting Started: Decide Which Interest‐Holders Should be Engaged

Cochrane review authors are encouraged to involve interest‐holders and should describe their engagement in the methods section. If the review is being co‐produced with interest‐holders, they are engaged before the question is developed. For health equity, authors should consider including people who have lived experiences of inequities to add the additional perspectives that would be important to improve the usefulness, relevance, uptake, and impact of the results of the review. Review authors should also engage with other interest‐holders (see Box 2) from the planning stages of the review through to its completion. These interest‐holders can help with many steps of the review process, such as defining the scope of the review, selecting outcomes, and interpreting the findings [13, 14, 15]. For information about engaging consumers see https://training.cochrane.org/online-learning/consumer-involvement.

Box 2 **Who are interest‐holders?**

Interest‐holders are groups with legitimate interests in the health issue being assessed [11]. They include: patients/consumers, caregivers, patient groups; payers or funders of research; payers or purchasers of health services; publishers and peer review journal editors; policymakers; principal investigators and research team members; product makers; producers and commissioners of evidence syntheses; program managers; providers; and members of the public [11]. These can be referred to as the 11 “Ps” [12].

You don't have to involve all these groups in your review, but you should consider which would be most relevant for your question.

Authors should report who was engaged, how they were recruited, and how and when they contributed to the review to ensure transparency.

**Example.** A review of interventions to address precarious housing, identified early on that people with lived experience of homelessness were very much needed to understand the complex and dynamic context in which the interventions would take place. They identified 4 persons with history of homelessness and provided them with basic training and also compensated them as advisors to prioritize interventions and outcomes and also help with the final wording of results. Pottie et al. [16].

## 5 Getting Started With Health Equity in Your Review

### 5.1 Consider Health Equity at the Question Formulation Stage

When developing the review question and writing the background section of a protocol or review, authors should explicitly consider what health equity means in the context of their review. Authors should reflect on where inequities exist and where their review can contribute meaningfully to addressing them. If the review is being co‐produced with interest‐holders, they will develop the question and provide insights into these considerations with the rest of the review team. If appropriate, authors should consider using a framework, such as PROGRESS‐Plus, to help guide the identification of characteristics that may contribute to health inequities (Appendix A).

**FIGURE 1:**
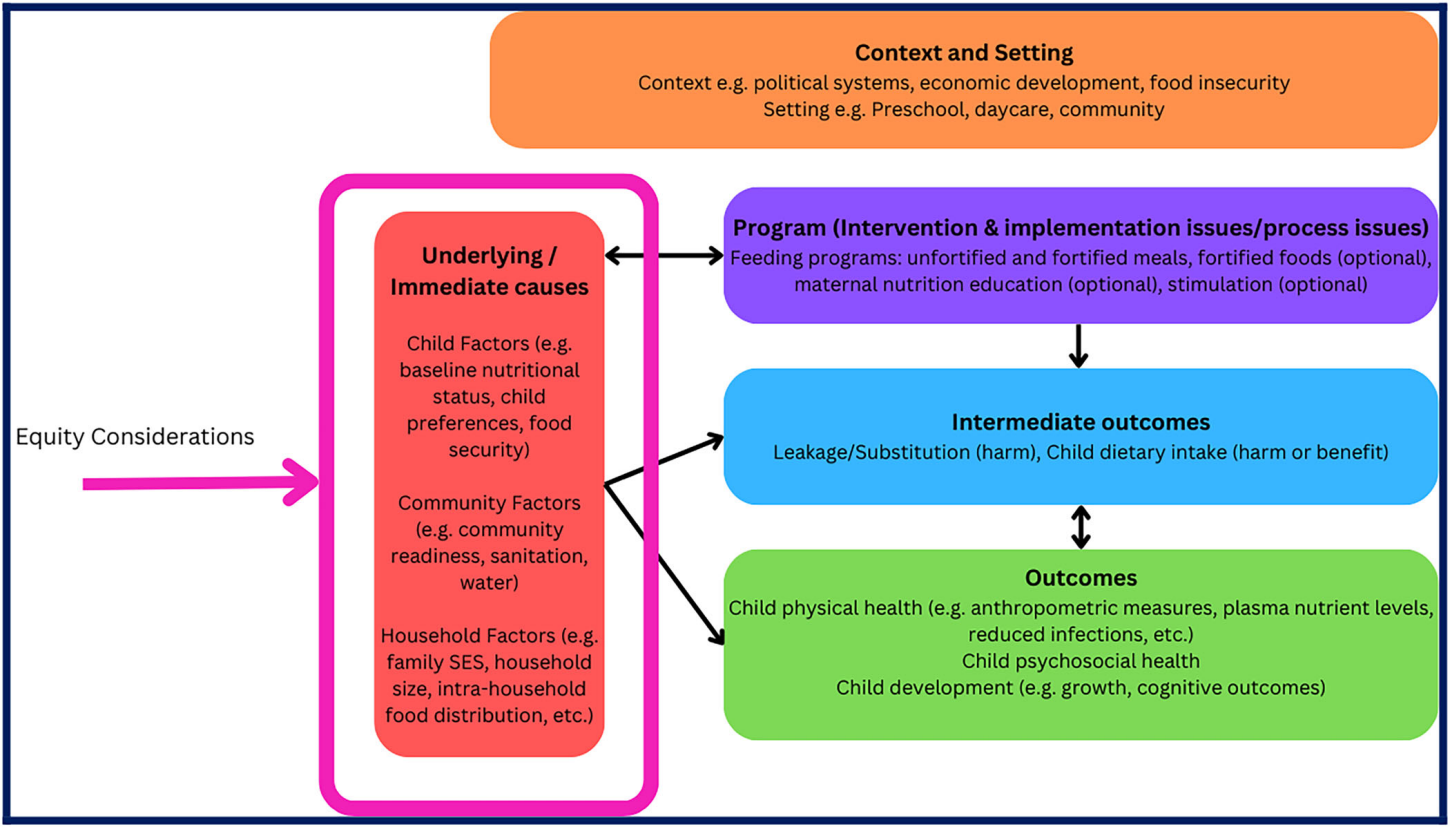
Logic model modified from [18], Food supplementation for improving the physical and psychosocial health of socio‐economically disadvantaged children aged 3 months to 5 years.

A logic model provides a visual description of the mechanisms or pathways in which an intervention is expected to work [17]. When applying a health equity lens, they can demonstrate the relationship between PROGRESS‐Plus factors and the delivery or receipt of the intervention as well as the effects of the intervention. Logic models may seem overwhelming, but they typically include standard components related to the inputs, processes, outcomes, and context [17]. The logic model should describe the impact of systemic sources of inequities such as racism, sexism, and other structural determinants. This should be followed by reflection on differences in lived experiences and baseline risks across population groups, and how these factors may influence the delivery, uptake, or effects of the intervention. Authors should describe any anticipated variations in intervention implementation or outcomes for the specified populations or settings. These steps should be informed by a range of evidence sources, including published literature, local or contextual data, lived experiences and the knowledge and perspectives of interest‐holders. See an example in Figure 1, where household factors such as socioeconomic status influence the implementation of the intervention. In this example, contextual factors, such as child factors, community factors, and household factors were expected to influence the intervention [18]. The authors of this review considered household factors and their possible effect on the intervention, such as food distribution within the household because of the possibility of substitution of food for those receiving the feeding program.

In the background section, authors should describe health equity using the same structured reasoning used to the develop the logic model—even if one was not formally developed. This includes how social structures, (e.g. racism, sexism, ageism), affect the condition of interest. Authors can describe whether there are differences in the lived experiences of the included populations that may affect the condition or intervention and whether there might be differences in the importance of outcomes to different populations.

Authors should consider whether there are differences in the baseline risk of the condition for some groups within the population or whether there are factors that would affect the effectiveness of the intervention. Authors should describe any expected differences in the implementation of, or outcomes of, the intervention for specific populations and define these populations and settings. Authors can utilize existing literature, local data, or lived experience to report this information (Box 3).

**Example.** A review assessing communication strategies to promote acceptance, uptake, and adherence to social distancing related to the COVID‐19 pandemic including the following “It is clear that inequalities influence the degree to which individuals and populations are able to accept and adhere to preventive measures. Accordingly, the importance of public communication that recognizes and is designed to counteract inequalities can't be overstated. This is critical to supporting community‐level uptake of physical distancing measures – particularly as the effects of the pandemic disproportionately affect the poorest and most vulnerable.” [20]

Box 3 **Common reasons for not considering health equity.**

***What if the included studies are lacking the relevant data?*** Authors are always encouraged to contact the primary study authors for more information, if needed, for their review [19]. If data are sought but ultimately unavailable, review authors can state what they would have liked to explore but were unable to because of a lack of data in the studies or because of a lack of resources. This will help inform future research and updates of the review.

***Including health equity considerations will take too much time.*** You're already extracting characteristics of the included studies. Include some information about the populations included within your studies, such as the age, sex, location, or other important details and include these in your characteristics of included studies or in a table summarizing across all included studies.

***We don't have the health equity expertise on our review author team***. There are resources to help you get started. See the section below on further reading and online content.

## 6 Ready for More Information About Including Health Equity in Your Review?

If you are ready to continue considering health equity throughout the methods and results of your review, please read part 2 of this tutorial.

Additionally, information and resources for including health equity in reviews are available on the Cochrane Training (https://training.cochrane.org/) and Cochrane Equity websites (https://methods.cochrane.org/equity/).

## 7 Further Reading and Online Content

We have developed a micro‐learning module, including a short quiz with feedback, to illustrate the common errors covered in this tutorial (https://share.gomolearning.com/sharelink/4a319ccc76cb3ca757a7d7f1a73b7ee908b74874fba01b12b4/). Additional material is also provided in the learning module “Health Equity in Systematic Reviews” developed by Cochrane Training. This can be found here: https://www.cochrane.org/learn/courses-and-resources/interactive-learning/module-11-health-equity-systematic-reviews.

## Author Contributions

**Jennifer Petkovic:** conceptualization, writing – original draft, writing – review and editing, methodology, project administration. **Jordi Pardo Pardo:** conceptualization, methodology, writing – review and editing. **Vivian Welch:** conceptualization, methodology. **Omar Dewidar:** writing – review and editing. **Lara J. Maxwell:** writing – review and editing. **Andrea Darzi:** writing – review and editing. **Tamara Lotfi:** writing – review and editing. **Lawrence Mbuagbaw:** writing – review and editing. **Kevin Pottie:** writing – review and editing. **Peter Tugwell:** writing – review and editing.

## Ethics Statement

The authors have nothing to report.

## Consent

The authors have nothing to report.

## 1 Peer Review

The peer review history for this article is available at https://www.webofscience.com/api/gateway/wos/peer-review/10.1002/cesm.70055.

## Data Availability

Data sharing not applicable to this article as no datasets were generated or analyzed during the current study.

## Appendix A PROGRESS‐Plus

**Table:**

Characteristic	Description
Place of residence	“Place” refers to where someone lives and includes whether they live in a city or a rural area or the country (e.g. high‐, middle‐, or low‐income) and the characteristics of the home environment, neighborhood, city, region, country
Race/ethnicity/culture/language/ancestry	Race is commonly associated with physical characteristics and may overlap with aspects of ethnicity, ancestry, culture, or language. These may also relate to where someone lives or their religion. These characteristics or classifications can affect health or opportunities for health
Occupation	Refers to the type of occupation that a person has, as well as whether they are out‐of‐work, unemployed, or under‐employed
Gender and sex	Gender is a social concept that refers to expected roles. Gender can impact access to health services as well as decision‐making power. Gender roles can also impact exposures to health hazards in the home, workplace, or environment
	Sex refers to biological differences which may not be considered health inequities as these differences may not be avoidable
Religion	This refers to the religious beliefs and can affect health equity when the choices related to these beliefs are imposed on a person by their family or community
Education	This refers to the level of education. Higher education is linked to better health, including healthier lifestyles, access to preventive health measures
Socioeconomic status	Refers to a person's socioeconomic status (SES). Having a higher SES often affects the social determinants of health and allow for better living conditions, greater access to nutritious foods, increased access to healthcare, and access to better education
Social capital	Social capital refers to social networks or communities
Plus – additional individual characteristics	Other characteristics that may contribute to health inequities that are not included in PROGRESS, such as age, sexual orientation
Plus – characteristics of relationships	Refers to relationships that may contribute to inequities
Plus – time‐dependent situations	Refers to situations in which a person may experience inequities temporarily

## References

[1] M. Whitehead , “The Concepts and Principles of Equity and Health,” International Journal of Health Services 22 (1992): 429–445. 1644507 10.2190/986L-LHQ6-2VTE-YRRN

[2] WHO Health Equity. 2025, accessed July 30, 2025 from https://www.who.int/health-topics/health-equity#tab=tab_1.

[3] WHO ., Operational Framework for Monitoring Social Determinants of Health Equity (World Health Organization, 2024).

[4] J. O'Neill , H. Tabish , V. Welch , et al., “Applying an Equity Lens to Interventions: Using Progress Ensures Consideration of Socially Stratifying Factors to Illuminate Inequities in Health,” Journal of Clinical Epidemiology 67, no. 1 (2014): 56–64, 10.1016/j.jclinepi.2013.08.005. 24189091

[5] S. Oliver , J. Kavanagh , J. Caird , T. Lorenc , K. Oliver , and A. Harden , “Health Promotion, Inequalities and Young People's Health,” A Systematic Review of Research, EPPI‐Centre Report No. 1611 (2008), 2, http://eppi.ioe.ac.uk/cms/LinkClick.aspx?fileticket=lsYdLJP8gBI%3d&tabid=2412&mid=4471&language=en-US.

[6] Combahee River Collective . Combahee River Collective Statement. Published online April 1977, accessed October 23, 2020. https://americanstudies.yale.edu/sites/default/files/files/Keyword%20Coalition_Readings.pdf.

[7] G. R. Bauer , “Incorporating Intersectionality Theory into Population Health Research Methodology: Challenges and the Potential to Advance Health Equity,” Social Science & Medicine 110 (2014): 10–17. 24704889 10.1016/j.socscimed.2014.03.022

[8] L. Bowleg , “The Problem With the Phrase Women and Minorities: Intersectionality—An Important Theoretical Framework for Public Health,” American Journal of Public Health 102 (2012): 1267–1273, 10.2105/AJPH.2012.300750. 22594719 PMC3477987

[9] K. W. Crenshaw , On Intersectionality: Essential Writings (The New Press, 2017).

[10] P. H. Collins , Intersectionality as Critical Social Theory (Duke University Press, 2019).

[11] E. A. Akl , J. Khabsa , J. Petkovic , et al., “‘Interest‐Holders’: A New Term to Replace ‘Stakeholders’ in the Context of Health Research and Policy,” Cochrane Evidence Synthesis and Methods 2 (2024): e70007. 40475280 10.1002/cesm.70007 PMC11795957

[12] P. Tugwell , V. Welch , O. Magwood , et al., “Protocol for the Development of Guidance for Collaborator and Partner Engagement in Health Care Evidence Syntheses,” Systematic Reviews 12 (2023): 134. 37533051 10.1186/s13643-023-02279-1 PMC10394942

[13] INVOLVE . Factors Affecting Public Engagement by Researchers: Reflections on the Changing Landscape of Public Engagement by Researchers in the UK 2015, https://wellcome.ac.uk/sites/default/files/wtp060034.pdf.

[14] J. Kreis , M. A. Puhan , H. J. Schünemann , and K. Dickersin , “Consumer Involvement iIn Systematic Reviews of Comparative Effectiveness Research,” Health Expectations 16, no. 4 (2013): 323–337. 22390732 10.1111/j.1369-7625.2011.00722.x PMC5060681

[15] J. Brett , S. Staniszewska , C. Mockford , et al., “A Systematic Review of the Impact of Patient and Public Involvement on Service Users, Researchers and Communities,” Patient ‐ Patient‐Centered Outcomes Research 7, no. 4 (2014): 387–395. 25034612 10.1007/s40271-014-0065-0

[16] K. Pottie , C. E. Kendall , T. Aubry , et al., “Clinical Guideline for Homeless and Vulnerably Housed People, and People With Lived Homelessness Experience,” Canadian Medical Association Journal 192, no. 10 (2020): E240–E254. 32152052 10.1503/cmaj.190777 PMC7062440

[17] L. M. Anderson , M. Petticrew , E. Rehfuess , et al., “Using Logic Models to Capture Complexity in Systematic Reviews,” Research Synthesis Methods 2 (2011): 33–42. 26061598 10.1002/jrsm.32

[18] E. Kristjansson , D. K. Francis , S. Liberato , M. Benkhalti Jandu , V. Welch , et al., “Food Supplementation for Improving the Physical and Psychosocial Health of Socio‐Economically Disadvantaged Children Aged Three Months to Five Years,” Cochrane Database of Systematic Reviews no. 3 (2015): CD009924, 10.1002/14651858.CD009924.pub2. 25739460 PMC6885042

[19] T. Li , I. J. Saldanha , J. Jap , et al., “A Randomized Trial Provided New Evidence on the Accuracy and Efficiency of Traditional vs. Electronically Annotated Abstraction Approaches in Systematic Reviews,” Journal of Clinical Epidemiology 115 (2019): 77–89. 31302205 10.1016/j.jclinepi.2019.07.005

[20] R. E. Ryan , C. Silke , A. Parkhill , A. Virgona , B. Merner , et al., “Communication to Promote and Support Physical Distancing for COVID‐19 Prevention and Control,” Cochrane Database of Systematic Reviews no. 10 (2023): CD015144, 10.1002/14651858.CD015144. 37811673 PMC10561351

